# Dutch patients, caregivers and healthcare professionals generate first nationwide research agenda for juvenile idiopathic arthritis

**DOI:** 10.1186/s12969-021-00540-2

**Published:** 2021-04-07

**Authors:** Anouk Verwoerd, Wineke Armbrust, Katherine Cowan, Lotte van den Berg, Joke de Boer, Sanne Bookelman, Marjan Britstra, Jeannette Cappon, Maria Certan, Christine Dedding, Karin van den Haspel, Petra Hissink Muller, Karin Jongsma, Otto Lelieveld, Jorg van Loosdregt, Wendy Olsder, Johanna Rocha, Ellen Schatorjé, Natasja Schouten, Joost F. Swart, Sebastiaan Vastert, Margot Walter, Casper G. Schoemaker

**Affiliations:** 1 Centre for Translational Immunology, University Medical Centre Utrecht, Heidelberglaan 100, 3584 CX Utrecht, The Netherlands; 2 Department of Paediatric Immunology and Rheumatology, Wilhelmina Children’s Hospital, Lundlaan 6, 3584 EA Utrecht, The Netherlands; 3 University of Groningen, University Medical Centre Groningen, Department of Paediatric Rheumatology and Immunology, Beatrix Children’s Hospital, Hanzeplein 1, 9713 GZ Groningen, The Netherlands; 4 Dutch Society for Paediatric Rheumatology (NVKR), Mercatorlaan 1200, 3528 BL Utrecht, The Netherlands; 5 James Lind Alliance, National Institute for Health Research Evaluation, Trials and Studies Coordinating Centre (NETSCC), based at the University of Southampton, Alpha House, Enterprise Road, Southampton, SO16 7NS UK; 6 Dutch JIA Patient and Parent Organisation (JVN), member of ENCA, Pius X-straat 49, 5121 EP Rijen, The Netherlands; 7 Department of Ophthalmology, University Medical Centre Utrecht, Heidelberglaan 100, 3584 CX Utrecht, The Netherlands; 8 Reade, Centre for Rehabilitation and Rheumatology, Dr. Jan van Breemenstraat 2, 1056 AB Amsterdam, The Netherlands; 9 Dutch Health Professionals in Paediatric Rheumatology (NHPKR), Amsterdam, The Netherlands; 10 Department of Science, University College Roosevelt, Lange Noordstraat 1, 4331 CB Middelburg, The Netherlands; 11 Department of Medical Humanities, Amsterdam University Medical Centre, De Boelelaan 1117, 1081 HV Amsterdam, The Netherlands; 12 Department of Paediatric Immunology and Rheumatology, Willem-Alexander Children’s Hospital, Albinusdreef 2, 2333 ZA Leiden, The Netherlands; 13 Julius Centre for Health Sciences and Primary Care, University Medical Centre Utrecht, Heidelberglaan 100, 3584 CX Utrecht, The Netherlands; 14 University of Groningen, University Medical Centre Groningen, Department of Rehabilitation Medicine, Hanzeplein 1, 9713 GZ Groningen, The Netherlands; 15 Youth-R-Well.com, Patient Organisation for Young Patients, member of EULAR PARE, Eikstraat 3, 3434 TD Nieuwegein, The Netherlands; 16 Department of Paediatric Immunology and Rheumatology, Amalia Children’s Hospital, Geert Grooteplein Zuid 10, 6525 GA Nijmegen, The Netherlands; 17 Department of Paediatric Rheumatology, St. Maartenskliniek, Dokter Kopstraat 1, 5835 DV Beugen, The Netherlands; 18 Department of Rheumatology, Erasmus University Medical Centre, Doctor Molewaterplein 40, 3015 GD Rotterdam, The Netherlands; 19 Faculty of Medicine, Utrecht University, Universiteitsweg 98, 3584 CG Utrecht, The Netherlands

**Keywords:** Juvenile idiopathic arthritis, Research priority setting, James Lind Alliance, Patient involvement

## Abstract

**Background:**

Involving the end-users of scientific research (patients, carers and clinicians) in setting research priorities is important to formulate research questions that truly make a difference and are in tune with the needs of patients. We therefore aimed to generate a national research agenda for Juvenile Idiopathic Arthritis (JIA) together with patients, their caregivers and healthcare professionals through conducting a nationwide survey among these stakeholders.

**Methods:**

The James Lind Alliance method was used, tailored with additional focus groups held to involve younger patients. First, research questions were gathered through an online and hardcopy survey. The received questions that were in scope were summarised and a literature search was performed to verify that questions were unanswered. Questions were ranked in the interim survey, and the final top 10 was chosen during a prioritisation workshop.

**Results:**

Two hundred and seventy-eight respondents submitted 604 questions, of which 519 were in scope. Of these 604 questions, 81 were generated in the focus groups with younger children. The questions were summarised into 53 summary questions. An evidence checking process verified that all questions were unanswered. A total of 303 respondents prioritised the questions in the interim survey. Focus groups with children generated a top 5 of their most important questions. Combining this top 5 with the top 10s of patients, carers, and clinicians led to a top 21. Out of these, the top 10 research priorities were chosen during a final workshop. Research into pain and fatigue, personalised treatment strategies and aetiology were ranked high in the Top 10.

**Conclusions:**

Through this study, the top 10 research priorities for JIA of patients, their caregivers and clinicians were identified to inform researchers and research funders of the research topics that matter most to them. The top priority involves the treatment and mechanisms behind persisting pain and fatigue when the disease is in remission.

## Background

Juvenile Idiopathic Arthritis (JIA) is a heterogeneous disease that is characterised by the occurrence of arthritis of unknown origin lasting for more than 6 weeks, with an onset before the age of 16 years. It is the most common chronic rheumatic disease in children [1]. The health outcome for children and young adults with JIA has significantly improved, however unanswered questions still remain regarding the care of these patients [1, 2]. Nevertheless, there is increasing evidence that the needs raised by patients with JIA, their caregivers and clinicians are not always reflected by the subjects as studied in research programmes [3]. Importantly, various studies demonstrate the importance of involving the end-users of knowledge in setting priorities for research to formulate research questions that truly make a difference and are in tune with the needs of patients [4, 5].

While the importance is widely acknowledged [6], still little effort is made to include patients in the set-up of JIA research. This is probably also aggravated by the fact that it includes a paediatric population, and it is still not exactly clear how to best include their voice [7–10]. A recent systematic review on research priority setting in paediatric chronic disease shows that only one in four studies reported parents/caregiver involvement, and only 5% included children directly [7]. One of the studies that directly involves children/adolescents in research priority setting for rheumatic conditions was a study by Parsons et al. They successfully involved young people (11–24 years) in research prioritisation by organising focus groups [11]. The domains ‘basic science’ and ‘psychosocial research’ were found to be most significant and have the highest priority for research. Notably, they showed that young people can discuss and prioritise scientific research, despite them being relatively research naïve. We aimed to interweave the advantages of such focus groups with the broader, well-established approach of setting research priorities of the James Lind Alliance (JLA).

The JLA is a non-profit initiative, founded in 2004 in the United Kingdom to bring patients, carers (caregivers, here: mostly parents), and clinicians together in Priority Setting Partnerships (PSPs) to set research priorities [12, 13]. They have developed a methodological approach that has now been used worldwide to generate about a hundred research agendas. Following the JLA methodology, we aimed to conduct a nationwide prioritisation exercise for JIA in the Netherlands [2]. The ultimate aim of this study is to guide future JIA research and funding to the issues that matter most to all directly involved.

## Methods

### Ethical considerations

The Medical Ethical Committee of University Medical Centre Utrecht confirmed that this study was exempted from the Medical Research Involving Humans Act (WMO) (METC protocol number 18–721/C).

### Set-up

**Fig. 1:**
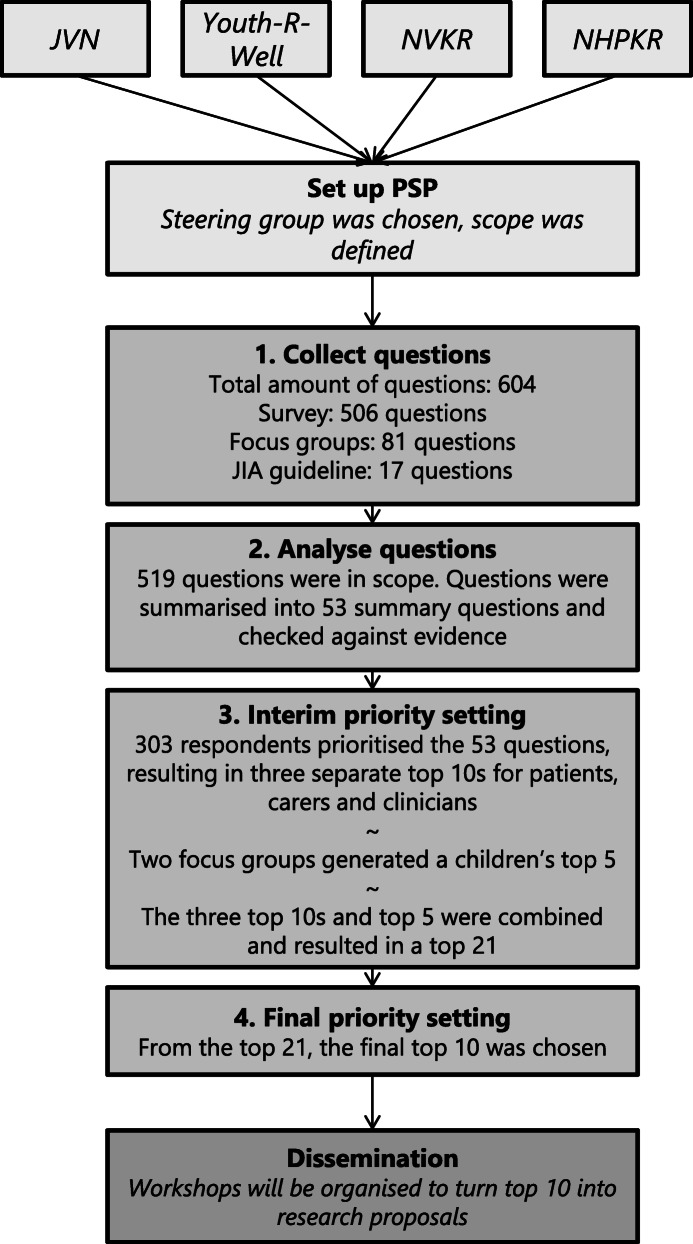
A flow chart of the priority setting process. This Priority Setting Partnership was initiated by the Dutch JIA patient and parent organisation (JVN),Youth-R-Well.com (an association for young people with JIA), NVKR (the Dutch Society for Paediatric Rheumatology), and NHPKR (Dutch Health Professionals in Paediatric Rheumatology). This chart demonstrates the steps that were taken to generate the research agenda

This PSP was conducted in the Netherlands. The full set-up is demonstrated in Fig. 1 and also described in a protocol article in this journal [2, 14].

#### Initiating parties and steering group

The PSP was initiated by two Dutch patient associations for JIA (the Dutch JIA patient and parent organisation (JVN) and Youth-R-Well.com (YRW)), and two Dutch professional organisations (the Dutch Society for Paediatric Rheumatology (NVKR), and the Dutch Health Professionals in Paediatric Rheumatology (NHPKR)). An expert steering group was assembled to oversee all phases of the project. This group consisted of young adult JIA patients (*n* = 4), parents of JIA patients (*n* = 3), paediatric rheumatologists (n = 3), an ophthalmologist (*n* = 1), physical therapists (*n* = 2) and a nurse practitioner (n = 1). Clinicians were recruited from 7 academic centres in the Netherlands. They were selected to represent all different professions and all geographic regions of the Netherlands. Most of them were members of the abovementioned organisations. Our JLA advisor (KC) had an advisory role throughout the whole process, while the steering group had the authority to make all important decisions within the framework and guiding principles of the JLA.

#### Scope

Before starting the process, the scope of our PSP was defined in agreement with the steering group. We chose to keep our scope broad to include questions regarding prevention, aetiology, symptoms, diagnosis, treatment, prognosis, health services, self-management and psychosocial aspects. We explicitly made the commitment to include lower educated carers and patients, and children to be truly inclusive.

#### Different phases

##### Collecting questions

The first step was to collect relevant questions from patients, carers and clinicians through a survey (December 2018–March 2019). Patients and parents were recruited to participate via patient organisations (e-mail and social media), and via flyers distributed at all outpatient clinics for paediatric rheumatology in the Netherlands. Clinicians were recruited via professional organisations’ newsletters and flyers were sent to them. Demographic details were analysed periodically to strive for equal representation of age, education level and region. Recent JIA guidelines were also searched for research recommendations. Extracted recommendations were added to the question database. Focus groups and small (group-) interviews were held with children (aimed 10–13 years) in an exploratory, conversation-like manner, guided by an expert in child participation (among others, CD). In-depth results of the focus groups will be published separately (Aussems et al., in preparation).

##### Analysing questions

All questions were reviewed and assessed whether they were in scope. Questions that were identified out of scope were put on a separate list which was agreed upon in a steering group meeting. Next, the included questions were grouped into categories and keywords were allocated to them, to ease the process of summarising. In a face-to-face meeting, the steering group summarised the questions into summary questions. A search in PubMed and PsycINFO (restricted to the last 10 years) was conducted to ensure that the question was unanswered. Publications were found to be relevant if they included systematic reviews, meta-analyses, randomised control trials (RCTs) or large representative cohort studies following the JLA guidelines. Next, the steering group provided expert opinion on the articles that were retrieved from this search and made the final decision about whether or not the question could be identified as already answered.

##### Interim priority setting survey

Here, the goal was to make a shortlist of 20–25 questions for the final workshop. Again, a survey was designed in which participants could choose their Top 10 (October–December 2019). Participants were recruited in the same way as during the first survey. Furthermore, participants of the first survey who left their contact details were e-mailed directly. Younger children were asked to rank their top 5 during focus groups that were set up as small priority setting workshops. Three separate top 10s, for patients, carers and clinicians, were determined. Together with the top 5 of the younger children, a final shortlist was set – they all had an equal weight. This shortlist was taken to the final workshop.

##### Final priority setting workshop

Participants of the workshop were invited from the steering group, through professional connections and through the interim survey (people who left their contact details were approached) to be inclusive of the whole JIA community. Prior to the workshop, participants were asked to individually prioritise the shortlist of questions. Through three rounds of prioritisation in both smaller and larger groups, chaired by trained facilitators using an adapted nominal group technique, the final top 10 was agreed (February 2020). Several observers were invited from a large patient advocacy organisation, an insurance company, the Dutch Society for Paediatrics (NVK) and the Dutch Arthritis Foundation. They did not take part in the discussions, but were present to observe the process, become inspired and learn from the discussions.

### Process evaluation

An independent ethical process evaluation was conducted by a bio-ethicist (KJ) in order to evaluate (the inclusiveness of) the priority setting process. The bio-ethicist was present during all steering group meetings, the final workshop, and was included in all correspondence regarding the research agenda. In-depth results of this evaluation will be published separately (Jongsma et al., in press).

## Results

### Initial survey

During the first phase of the process, a total of 604 questions were gathered. Of these 604, 506 were submitted by 278 respondents through the survey: 141 patients (50.7%), 88 carers (31.6%) and 49 health care professionals (17.6%). Demographic data are summarised in Table 1. Responses were gathered from all academic centres and geographic regions in the Netherlands. In the online survey, 25.8% of the carers had finished a lower and middle education. To lower the bar of participating, a hardcopy version of the survey was distributed at outpatient clinics. On this hardcopy version 59% of the respondents had lower and middle education (see Table 2), demonstrating the additional value of distributing hardcopy surveys. The underrepresentation of younger patients in the online survey – no more than 23% of responding patients were younger than 16 years – underscored the significance of the focus groups. In total 24 children (17 girls and 7 boys, 9–16 years of age, suffering from JIA between 4 months to 9 years), participated in two focus groups and six (group-) interviews, Together, they formulated 81 extra questions (Aussems et al., in preparation). Furthermore, in the Dutch JIA guideline 17 additional unanswered research questions were found [15].

**Table 1:** Demographics of participants of the first and second survey

	First survey			Second survey		
**Respondents (number; %)**	Total	278 (100%)		Total	303 (100%)	
	Patients	141 (50.7%)		Patients	125 (41.3%)	
	Carers	88 (31.6%)		Carers	136 (44.9%)	
	Clinicians	49 (17.6%)		Clinicians	42 (13.9%)	
**Level of education of respondent**^a^ **(number; %)**	Patients	Primary school	22 (15.6%)	Patients	Primary school	3 (2.4%)
		PSE	21 (14.9%)		PSE	17 (13.6%)
		SGSE	13 (9.2%)		SGSE	22 (17.6%)
		PUE	19 (13.5%)		PUE	15 (12.0%)
		SVE	26 (18.4%)		SVE	25 (20.0%)
		HPE	20 (14.2%)		HPE	18 (14.4%)
		University	13 (9.2%)		University	20 (16.0%
		Other	7 (5.0%)		Other	5 (4.0%)
	Carers	Primary school	5 (5.7%)	Carers	Primary school	13 (9.6%)
		PSE	5 (5.7%)		PSE	6 (4.4%)
		SGSE	4 (4.5%)		SGSE	12 (8.8%)
		PUE	2 (2.3%)		PUE	2 (1.5%)
		SVE	14 (15.9%)		SVE	38 (27.9%)
		HPE	43 (48.9%)		HPE	47 (34.6%)
		University	12 (13.6%)		University	12 (8.8%)
		Other	3 (3.4%)		Other	6 (4.4%)
**Age of responding patient (mean; range)**	mean 17.6 (range 4–55) years			mean 19..95 (range 10–52) years		
**JIA subtype of patient (number; %)**	Polyarticular JIA	93 (40.6%)		Polyarticular JIA	110 (42.3%)	
	Oligoarticular JIA	57 (24.8%)		Oligoarticular JIA	50 (19.2%)	
	Psoriatric arthritis	10 (4.4%)		Psoriatric arthritis	15 (5.7%)	
	Enthesitis-related arthritis	2 (0.9%)		Enthesitis-related arthritis	5 (1.9%)	
	Systemic JIA	18 (7.9%)		Systemic JIA	31 (11.9%)	
	Don’t know	49 (21.4%)		Don’t know	50 (19.2%)	
**Disease duration in years** **(mean; range)**	8.8 years (0–65 years)			9.0 years (0–45 years)		
**Uveitis (number; %)**	Yes	51 (22.2%)		Yes	66 (25.3%)	
	No	174 (76.0%)		No	189 (72.4%)	
	Don’t know	4 (1.7%)		Don’t know	6 (2.3%)	
**Location of treatment centre (number; %)**	Groningen	72 (25.9%)		Groningen	48 (15.8%)	
	Utrecht	49 (17.6%)		Utrecht	51 (16.8%)	
	Nijmegen	10 (3.6%)		Nijmegen	5 (1.7%)	
	Boxmeer	29 (10.4%)		Boxmeer	31 (10.2%)	
	Amsterdam (Reade)	15 (5.4%)		Amsterdam (Reade)	13 (5.0%)	
	Amsterdam (UMC)	10 (3.6%)		Amsterdam (UMC)	13 (5.0%)	
	Leiden	10 (3.6%)		Leiden	18 (6.9%)	
	Rotterdam	79 (28.4%)		Rotterdam	82 (27.1%)	
	Maastricht	1 (0.4%)		Maastricht	5 (1.7%)	
	Other	3 (1.1%)		Other	37 (12.2%)	

^a^ Levels of education: *PVSE* Pre-vocational secondary education (in Dutch: VMBO), *SGSE* Senior general secondary education (in Dutch: HAVO), *PUE* Pre-university education (in Dutch: VWO), *SVE* Secondary vocational education (in Dutch: MBO), *HPE* Higher professional education (in Dutch: HBO)

**Table 2:** Levels of education of carers responding to online vs. hardcopy versions of both surveys

	Online	Hardcopy	Total
**FIRST SURVEY**			
**Education level**^a^			
Lower (n, %)	4 (6.1%)	6 (27.2%)	10 (11.4%)
Middle (n, %)	13 (19.7%)	7 (31.8%)	20 (22.7%)
Higher (n, %)	47 (71.2%)	8 (36.3%)	55 (62.5%)
Other (n, %)	2 (3.0%)	1 (4.5%)	3 (3.4%)
*Total (n)*	*66*	*22*	*88*
**SECOND SURVEY**			
**Education level**^a^			
Lower (n, %)	16 (17.2%)	3 (7.0%)	19 (14.0%)
Middle (n, %)	31 (33.3%)	21 (48.8%)	52 (38.2%)
Higher (n, %)	43 (46.2%)	16 (37.2%)	59 (43.4%)
Other (n, %)	3 (3.2%)	3 (7.0%)	6 (4.4%)
*Total (n)*	*93*	*43*	*136*

^a^Lower indicates primary school, VMBO/MAVO (lower general secondary education). Middle indicates HAVO (higher general secondary education), VWO (A-levels/pre-university education) and MBO (intermediate vocational education). Higher indicates HBO (higher vocational education) and university

### Analysis of evidence uncertainties

Of the 604 submitted questions, 519 were determined to be in scope. Out of scope questions mostly related to logistics of care (e.g. “Why can’t blood be drawn at the GP’s office instead of at the hospital?”, or: “Do we have to pay for orthopaedic shoes ourselves or does the insurance cover it?”) or personal situations (e.g. “Which type of JIA do I have?”) and could not be answered by research. During a face-to-face meeting, the steering group summarized the remaining questions into 53 summary questions.

An elaborate search in PubMed and PsycInfo showed that none of the 53 questions was completely answered. A full list of all the submitted questions, the 53 summary questions, our search strategy for evidence checking and the critical appraisal of the studies has been published online [16].

### Interim survey

Three hundred and three people chose their top 10 from the 53 questions: 125 patients (41.3%), 136 carers (44.9%) and 42 clinicians (13.9%). Two focus groups with seven and nine children respectively (in total 9 girls and 7 boys, 10–15 years), and a subsequent group priority setting discussion attended by six girls and five boys from the focus groups, resulted in a top 5 of their most important questions (Aussems et al. in preparation).

The 35 questions in the respective top 10s of patients, carers, clinicians, and the top 5 of younger children showed considerable overlap. One question – on fatigue – was prioritised by all 4 groups. Four questions were ranked in three top 10s. This resulted in a combined top 21. Table 3 shows how these 21 questions were ranked in the different groups. Interestingly, the question “How can JIA be cured?” was highly prioritised by patients and carers, and ranked very low by the clinicians. The clinicians may have been less optimistic about the chances to find a cure in the near future. Three questions in the childrens’ top 5 – numbered 14, 16 and 19 in Table 3 – were ranked much lower by the other three groups. This illustrates the added value of the focus groups.

**Table 3:** Interim rankings of the Top 21 research questions and the rankings per group

No.	Question	Ranking patients	Ranking carers	Ranking clinicians	Ranking children
1.	Pain and fatigue are often present when the disease is in remission. How does this happen, what can one do about it, and can one predict which patients will suffer from them?	3	10	1^a^	
2.	What is the best treatment plan for each individual patient? (e.g. start a biological directly, which one, and what to do when the first one does not work *and how can medication best be tapered off?* ^b^*)*	42	28	7^a^	
3.	What is the best treatment plan for uveitis in JIA, and are there factors that predict its effectiveness?	36	25	5	
4.	Why are children with JIA fatigued more quickly, what can be done about it, and how can one best cope with the fatigue in daily life?	6^a^	6	1^a^	2
5.	How does JIA develop and which factors influence this?	6^a^	5	24	
6.	How can the course (flares, extensions, cure) of JIA be better explained and predicted?	15	9	6	
7.	What is the influence of nutrition on JIA, and can a diet help?	2	2	7^a^	
8.	What are the short and long term side effects/consequences of the drugs taken for JIA?	8	1	10^a^	
9.	What is the influence of JIA on future opportunities regarding school results, work and relationships?	9	11	20	1
10.	What is the influence of sports and exercise on JIA and vice versa?	24	37	7^a^	
11.	What are the long term physical consequences of JIA?	1	3	10^a^	
12.	How can JIA be cured?	4	4	42^a^	
13.	Is there an association between JIA and other (autoimmune) diseases, and if yes, how can one better understand this?	10^a^	8	42^a^	
14.	How can pain best be recognised and be treated (with medication), and what action can a patient take him/herself?	32	30	29^a^	3
15.	Which knowledge and skills are needed for patients and parents to achieve a healthy and active lifestyle?	38	24	4	
16.	How can pills be manufactured in such a way that they are easy to take? (i.e. shape, color, taste)	29	40	29^a^	4
17.	How can children/adolescents with JIA can best be supervised regarding school/education in order to minimize drop-out rates and absenteism?	10	15	10^a^	
18.	Is JIA inheritable, and if yes, in what way?	5	13	51	
19.	What is the best way to practice your favorite sport safely?	43	49	17	5
20.	Are there any strategies in alternative medicine that can help alleviate health complaints of JIA?	21	7	42^a^	
*21.*^b^	*When and how can medication for JIA best be tapered off?*	*37*	*21*	*3*	

^a^ Signifies that a question was ranked in joint place with another question

^b^ Question 21 was merged into question 2 during the final workshop

### Final workshop

During the final priority setting workshop, on February 7, 2020, the top 10 was chosen (see Table 3). The workshop was attended by five patients, five parents of patients, and ten clinicians (paediatric rheumatologists (*n* = 3), an ophthalmologist (*n* = 1), physical therapists (*n* = 2), nurses (*n* = 3), and a psychologist (*n* = 1)). Clinicians were recruited from all academic centres in the Netherlands. The results of two rounds of discussions and ranking in three mixed groups were combined, and discussed further in a final session. The participants chose to merge the two summary questions on personalised medicine and strategies to taper off medication (see Table 3). All 20 attendees unanimously agreed on the final Top 10.

The result of the discussions in the final workshop broadly reflected the interim rankings. For both patients and carers, 6 of the interim top 10 questions (60%) were selected. For clinicians it was 80%. For the focus groups 2 out of 5 questions (40%) were part of the final top 10. Importantly, question 9 of the final top 10 made it into the list because a young adult patient advocated for it during the final discussion, using the argument that it was the children’s top priority. Evaluations from the participants of the final workshop were very positive: patients, carers and clinicians felt like their voice was heard, that all voices mattered equally, and that the atmosphere was very positive.

## Discussion

This project has brought patients, caregivers and clinicians together in creating a research agenda for JIA using the JLA method. This is one of the first times a PSP has integrated the JLA approach with additional focus groups with children to ensure involvement of paediatric patients of all age categories. We found that the number one research priority involved the cause and care of pain and fatigue when the disease was in remission. This is in line with a qualitative study by Bromberg et al. that finds self-reported pain and fatigue are highly common in children with JIA despite advances in treatment strategies [17]. It is also highlighted to be an important research area by Palman et al. [18], along with defining better predictors of remission states, which was also part of our Top 10. The fact that these studies all underline the same top research priority demonstrates the importance of future research focusing on this. Next to pain and fatigue, the aetiology of the disease remains an important topic that was featured high in our Top 10 and also in a recent priority setting exercise in the United States [19].

Moreover, our study exposed other important research areas such as personalised treatment strategies, JIA-associated uveitis, nutrition, long-term effects of drugs taken for JIA and sports/exercise that were not directly featured in other studies. This may be due to the wide scope we defined for this project, as well as the easily accessible nature of the JLA method that may have led to the collection of more widespread themes. The fact that the questions do not only cover the healthcare setting but also comprise more (psycho) social and educational issues suggests funding for these topics can also be sought from psychological, social and educational funding bodies.

One major strength of our study is the use of the systematic and transparent JLA method, which enhances the validity of our results. Furthermore, our efforts to include younger children is a strength. We conducted focus groups during the two phases of the process where information was gathered, such that they truly had their say in determining the most important topics. Two questions of the children’s Top 5 made it to the final Top 10 (see Table 3), which indicates the significant influence they had in the PSP.

A challenge in this study was the inclusiveness of people with all educational backgrounds and age groups throughout the survey phases. The input of patients and parents in the steering group was very valuable. For example, they thought of using a cartoon on the flyers and in social media coverage to capture people’s attention. A parent proposed the use of hardcopy questionnaires in these phases, which improved the inclusion of respondents with regard to education level, especially in the first phase. For people with low digital skills, it might be more difficult to formulate their own question, than to choose from a list of questions.

In a subgroup analysis of the parent responses in the prioritising phase, we found that the online and hardcopy groups of parents showed overlap in their priorities. The parents responding with paper and pencil did not rank the question on alternative medicine that high. Interestingly, they did prioritize two questions on school and education (nrs 9 and 17), that were not prioritized in the online parent group. This may have been related to their own educational background.

In this light, it is important to realise that participants of the final workshop consisted of a group of (predominantly) white, articulate, higher educated people, despite explicit efforts to include people with lower educational backgrounds and other ethnicities. This may have influenced which questions made it to the Top 10. This is a typical tendency in survey research and was also observed in other PSPs [20]. Future PSPs may want to come up with and employ alternative strategies to ensure equal representation of all members of society. We also observed that more questions from the clinicians initial shortlist made it into the final Top 10 (see Table 3). This may be due to the fact that clinicians are more trained to give their opinion about these matters than young people and parents. Nevertheless, all attendees at the final workshop in the end unanimously agreed on the final Top 10 as a list of shared priorities.

Formally, this Top 10 was generated in the Netherlands, but we expect it also holds for other western countries. Now that the research agenda has been set, it is important that it is now indeed implemented with research funders and researchers. To achieve this, we attempted to inform the whole JIA research community in the Netherlands of this prioritisation exercise from the start, and some of the research groups made the commitment to truly incorporate the Top 10 in the long-term vision of their research lines. In addition, we have organised workshops with researchers, patients, parents and clinicians to turn the relatively broad questions into research proposals that can be presented to funding agencies. These workshops took place in the fall of 2020. With these workshops, we hope to gather a group of people that is truly committed to finding answers to the research questions of the Top 10.

## Conclusions

In short, we systematically and transparently generated a research agenda for JIA, confirmed as evidence uncertainties and regarded important by children and young people with JIA, their parents/caregivers and the clinicians caring for them. This is a vital resource that conveys a clear message to both government funders and charitable agencies, about which research topics should have priority. The next step is to turn the questions into research proposals. Workshops are currently being organised to this end.

## Data Availability

Most of the data described in the article can be found in this article or online at the James Lind Alliance website, as stated in the article and mentioned in the references. Any other datasets are available from the corresponding author on reasonable request.

## Abbreviations

JIA: Juvenile Idiopathic Arthritis
JLA: James Lind Alliance
JVN: Dutch JIA Patient and Parent Organisation
NHPKR: Dutch Health Professionals in Paediatric Rheumatology
NVK: Dutch Society for Paediatrics
NVKR: Dutch Society for Paediatric Rheumatology
PSP: Priority Setting Partnership
RCT: Randomised Controlled Trial
WMO: Medical Research Involving Humans Act
YRW: Youth-R-Well.com
